# Indoleamine 2,3 Dioxygenase (IDO) Expression and Activity in Relapsing- Remitting Multiple Sclerosis

**DOI:** 10.1371/journal.pone.0130715

**Published:** 2015-06-25

**Authors:** Roberta Mancuso, Ambra Hernis, Simone Agostini, Marco Rovaris, Domenico Caputo, Dietmar Fuchs, Mario Clerici

**Affiliations:** 1 Don C. Gnocchi Foundation ONLUS, Piazza Morandi 3, 20100, Milan, Italy; 2 Division of Biological Chemistry, Biocenter, Innsbruck Medical University, Innrain 80–82, A-6020, Innsbruck, Austria; 3 Department of Physiopathology and Transplantation, University of Milano, Via Fratelli Cervi 93, 20090, Segrate, Milano, Italy; University Hospital of Heidelberg, GERMANY

## Abstract

**Background:**

Interferon gamma (IFN-γ) production induces the transcription of indoleamine 2,3 dioxygenase (IDO) resulting in the reduction of T-cell activation and proliferation through the depletion of tryptophan and the elicitation of Treg lymphocytes. IDO was shown to be involved in the pathogenesis of autoimmune diseases; we investigated whether changes in IDO gene expression and activity could be indicative of onset of relapse in multiple sclerosis (MS) patients.

**Methods:**

IDO and interferon-γ (IFN-γ) gene expression, serum IDO activity (Kynurenine/Tryptophan ratio) and serum neopterin concentration – a protein released by macrophages upon IFN-γ stimulation – were measured in 51 individuals: 36 relapsing remitting (RR)-MS patients (21 in acute phase -AMS, 15 in stable phase -SMS) and 15 healthy controls (HC). PBMCs samples in AMS patients were collected before (BT-AMS) and during glucocorticoids-based therapy (DT-AMS).

**Results:**

IDO expression was increased and IFN-γ was decreased (p<0.001) in BT-AMS compared to SMS patients. Glucocorticoids-induced disease remission resulted in a significant reduction of IDO and IFN-γ gene expression, IDO catalytic activity (p<0.001). Serum neopterin concentration followed the same trend as IDO expression and activity.

**Conclusions:**

Measurement of IDO gene expression and activity in blood could be a useful marker to monitor the clinical course of RR-MS. Therapeutic interventions modulating IDO activity may be beneficial in MS.

## Introduction

Multiple Sclerosis (MS) is a chronic demyelinating neurodegenerative disorder of the central nervous system (CNS). This disease is characterized by perivascular inflammatory lesions in the white matter of the CNS, demyelination, and axonal damages and is probably initiated by autoreactive T lymphocytes directed toward CNS antigens. Activatory events occurring in the periphery, as well as failure in multiple regulatory mechanisms of immune tolerance, can foster the CNS infiltration of activated autoreactive T cells, resulting in tissue damage [1]. Relapsing remitting MS (RR-MS), the most common course of the disease, is characterized by periods of worsening neurologic function (relapse) followed by phases of partial or complete recovery (remissions). The molecular processes that lie behind the succession of these phases are only partially known.

Indoleamine 2,3 dioxygenase (IDO) is an enzyme that catalyses the first and rate-limiting step in the kynurenine (Kyn) pathway, *i*.*e*. the degradation of the essential amino acid tryptophan (Trp) into Kyn in extrahepatic tissues. This pathway plays a role in the pathogenesis of neuroinflammatory and neurodegenerative disorders. Thus, the intermediate metabolites of Trp catabolism can modulate neurotransmitter (serotonin, melatonin) and inflammatory pathways in peripheral and central nervous systems, where they can have both neurotoxic and neuroprotective roles [2]. IDO, in particular, plays a dual function (reviewed in [3]): on one hand it contributes to innate host defences as it directly inhibits the growth of microorganisms, on the other hand it promotes immune tolerance, facilitating the maturation of naive T lymphocytes into T regulatory cells, and increasing T cell susceptibility to apoptosis. Notably, Kyn is also endowed with immune properties as it stimulates the development and the activation of Treg cells [4] and, as a consequence, plays a role in tolerance and immune suppression.

Both IDO and Kyn were shown to be involved in oncogenesis, viral infections, and metabolic diseases. Thus, IDO over-expression in cancer [5] and in HIV disease [6] is associated with poor prognosis, whereas glycemic parameters worsen when IDO activity is blocked in diabetic patients [7]. The Kyn pathway, finally, is activated during neuroinflammation and seems to be implicated in different neurological conditions, including HIV-associated neurocognitive disorder, as well as Parkinson’s and Alzheimer’s diseases [8].

Interferon-γ (IFN-γ) stimulates IDO gene transcription in many cell types including macrophages, dendritic cells and fibroblasts [9]. This also takes place in the CNS, as the microglia expresses IDO upon treatment with IFN-γ [10, 11]. Recent evidences obtained in experimental autoimmune encephalomyelitis (EAE), the murine model of MS, show that IDO induction results in the down-modulation of neuroinflammation and in an improvement of the disease [12, 13]. Moreover, an opposing pattern of IDO and IFN-γ mRNA expression was observed in spinal cord and peripheral blood mononuclear cells (PBMCs) through the preclinical, acute and remission phases of EAE [12], suggesting that IDO and Trp metabolites contribute to the reversible neurological symptoms of MS. Nevertheless, although Trp levels and IDO activity have been repeatedly studied in MS patients [14–16], consistent results have not been obtained, probably due to the variable course of the disease.

In the attempt to shed light on the role of the IDO pathway in MS we analyzed IDO, IFN-γ, and IDO enzyme activity in PBMCs of RR-MS patients undergoing clinical relapse before and after the initiation of glucocorticoids (GC) therapy.

## Materials and Methods

### Patients and controls

Fifty-one individuals were enrolled in the study: 36 patients with a diagnosis of RR-MS and 15 age- and sex- matched healthy controls (HC). All patients were diagnosed as having relapsing remitting MS according to revised McDonald classification [17]; HC included laboratory and hospital staff with no history of autoimmune disease. MS patients in an acute phase of the disease (AMS, n = 21)—defined as an episode of neurological disturbance lasting for at least 24 hours and for which there was no other causes [17]—underwent clinical examination before and after GC treatment with intravenous methylprednisolone (1g/die for 5 days administered in two hours in the morning). The GC treatment was administered as standard care for the patients in this study. MS patients in a stable phase (SMS, n = 15) of disease were enrolled when free of treatment and no new neurological symptoms had been registered for at least two months before the inclusion in the study. Stable disease was diagnosed on the basis of brain and spinal cord magnetic resonance imaging (MRI) with gadolinium showing no areas of enhancement at the time of enrolment. The study conformed to ethical principles of the Helsinki Declaration and was approved by the local ethics committee of the Don C. Gnocchi Foundation ONLUS; all patients and controls gave their written informed consent to participate in the study.

### Sample collection and RNA extraction

RNA was extracted from PBMCs after whole blood collection, while sera were stored at -20°C. In AMS subjects, a blood sample was collected immediately before the first dose of GC (BT-AMS) as well as after 2 days of treatment (DT-AMS). In 8 patients blood samples were collected after one day of treatment as well and in 3 more cases, samples were also collected after four days of therapy. Total RNA was extracted using the RNAeasy Mini extraction kit (Qiagen, Hilden, Germany) and eluted in RNAse-Free water. Total RNA concentration was determined by spectrophotometer (OD: 260 nm). Purity was determined as the 260 nm/280 nm OD ratio with expected values between 1.8 and 2.0. RNA was treated with TURBO DNA-free DNAse (Ambion INC, Austin, TX, USA) and retrotranscribed with High-capacity cDNA Reverse Transcription Kit (Applied Biosystems, Foster City, CA, USA), as specified by manufacturers. cDNA samples were stored at –20°C until use.

### Relative quantitative real-time PCR (q-PCR)

Q-PCR experiments were performed on ABI Prism 7000 Sequence Detection System (Applied Biosystems), using pre-designed and validated primers (Applied Biosystems) for two housekeeping genes (hkg) (GAPDH assay IDs: Hs99999905_m1, ACTB Hs99999903_m1) and IDO (assay ID: Hs00984148_m1) and IFN-γ genes (assay ID: Hs00989291_m1) as targets.

Briefly, 2 μl of cDNA were used in a PCR reaction volume of 20 μl, containing 10 μl of Gene Expression Master Mix (Applied Biosystem), 1μl of TaqMan probe and 7 μl of water. PCR cycles were as follows: 10 min at 95°C followed by 40 cycles of 15°C at 95°C and 1 min at 60°C. All reactions were performed in triplicate, with non-template control for each gene. For each sample, relative gene expression of the target mRNA was calculated, relative to an endogenous reference gene (ΔCq_sample_: Cq_target_- Cq_hkg_). Gene-expression levels are given as normalization ratio calculated as: fold = 2^-[ΔCq(sample)-ΔCq(control)]^ [18].

### Sera measurements

Free Trp and Kyn serum concentrations (μM) were determined by high-performance liquid chromatography (HPLC) [19]. The ratio of Kyn/Trp (mmol/mol) was calculated as index of Trp breakdown. Neopterin concentrations (nM) were measured by an enzyme-linked immunosorbent assay (BRAHMS Diagnostics, Berlin, Germany).

### Statistical analysis

All data were normally distributed; they are reported as mean ± standard deviation (SD), whereas mRNA expression is reported as median of folding and interquartile range (IQR). As the main goal of this study was to compare AMS and SMS patients, SMS patients were used as reference group for mRNA folding measurement. Analyses of RNA relative expression were performed with the REST 2009 Software (http://www.qiagen.com/Products/REST2009Software.aspx?r=8042), that permits to use multiple reference genes for normalization, improving the reliability of results [20]. The expression ratio results are tested for significance by a Pair Wise Fixed Reallocation Randomisation Test.

Data of IDO activity were analyzed with ANOVA test and, when appropriate, with the Student’s t-test.

## Results

### Clinical outcome

Demographic and clinical data of the individuals included in the study are shown in Table 1. MS patients did not differ for disease duration or for EDSS indexes. Disease remission was achieved by GC in all AMS patients.

**Table 1:** Demographic and clinical data of the individuals enrolled in the study.

	AMS	SMS	HC
**N**	21	15	15
**Gender (M:F)**	8:13	5:10	5:10
**Age (years)**	36.29±9.78	40.25±5.92	37.83±9.55
**Disease duration (years)**	10.66±8.15	13.43±10.41	---
**EDSS**	3.15±1.37	4.14±1.75	---

Data are expressed as mean ± standard deviation. AMS: multiple sclerosis patients during disease relapse; SMS: multiple sclerosis patients in a stable phase of disease; HC: healthy controls; EDSS: expanded disability status score; SD: standard deviation.

### IDO and IFN-γ mRNA expression

**Fig 1:**
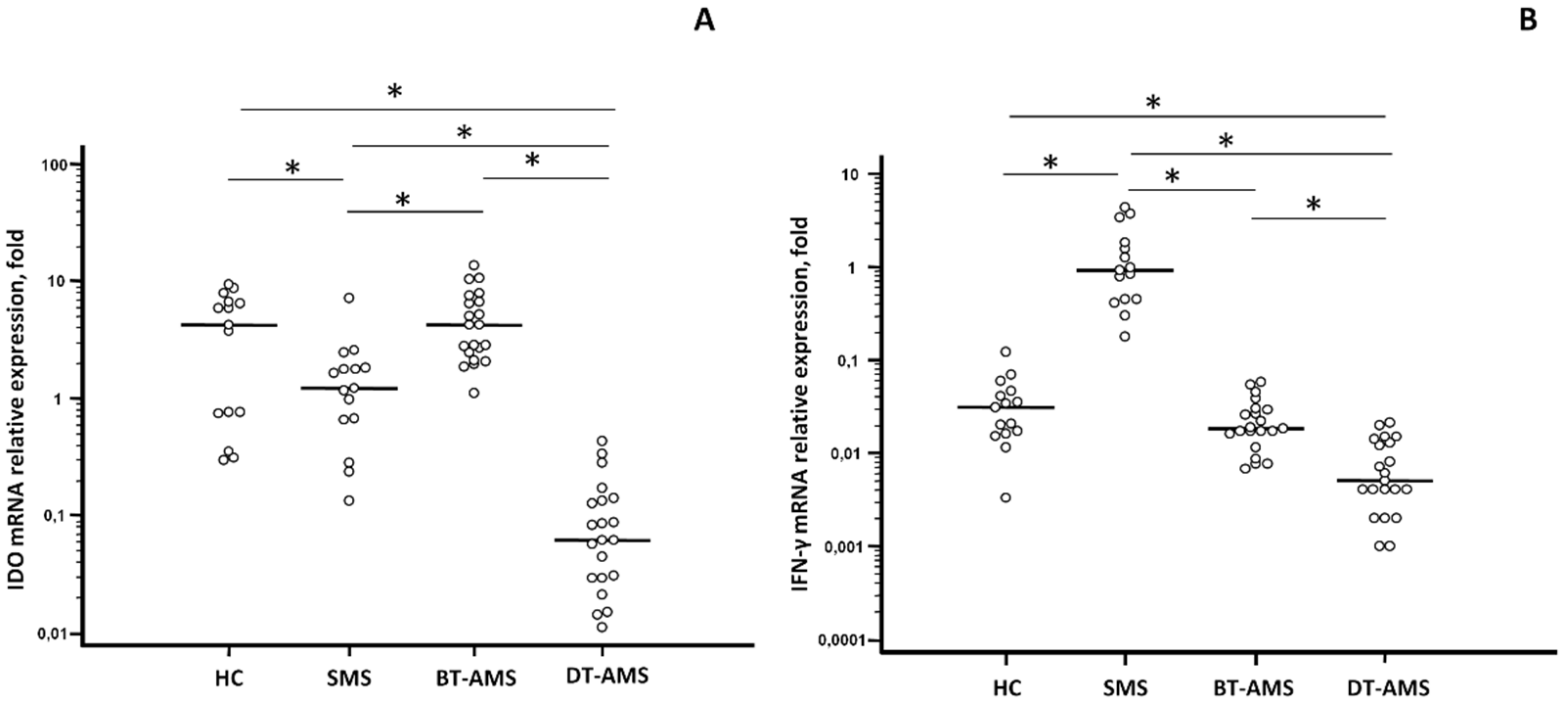
IDO and IFN-γ gene expression. mRNA relative expression of IDO (A) and IFN-γ (B) in healthy controls (HC) and in multiple sclerosis patients with stable (SMS) or relapsing (AMS) disease. AMS patients were analyzed before (BT-AMS) or after the initiation of glucocorticoid treatment (DT-AMS). *p<0.05.

IDO and IFN-γ mRNA expression was measured by q-PCR in PBMCs of SMS, AMS and HC individuals. AMS patients were studied before (BT-AMS) and after the initiation of GC therapy (DT-AMS). Results showed that, IDO expression was decreased (p = 0.01) (Fig 1, Panel A and Table 2) whereas that of IFN-γ was increased (p<0.001) (Fig 1, Panel B and Table 2) in SMS compared to HC. The expression of IDO was also significantly augmented in BT-AMS compared to SMS patients (p<0.001); this was drastically modified by therapy. Thus, GC-associated disease remission was correlated with a significant reduction of IDO expression (BT-AMS *vs*. DT-AMS p<0.001; DT-AMS *vs*. SMS and *vs*. HC p<0.001). Notably, IFN-γ expression was reduced in BT-AMS (p<0.001) compared to SMS, and decreased even more in DT-AMS (p = 0.01).

**Table 2:** IDO gene expression and enzymatic activity and neopterin concentration in patients and healthy controls.

	SMS	BT-AMS	DT-AMS	HC
**IDO (fold)**	1* ^#^ ^¶^	4.19	0.06	4.27
		(2.34–6.90)	(0.03–0.13)* ^¶^	(0.75–6.57)
**IFN-γ (fold)**	1* ^#^ ^¶^	0.02	0.005	0.03
		(0.01–0.03)	(0.003–0.13)* ^¶^	(0.02–0.05)
**Kyn (μM)**	2.48±0.51^#^	2.40±0.63^#^	1.85±0.35	2.65±.78^#^
**Trp (μM)**	78.80±14.11	73.69±11.63	76.89±10.77	69.21±16.40
**Kyn/Trp (mmol/mol)**	32.11±8.25^#^	34.52±11.78^#^	24.40±5.32*	38.75±9.37
**Neopterin (μM)**	4.81±1.12	5.02±1.64	4.70±1.10	5.69±3.15

Data are expressed as mean ± standard deviation or as median and IQR percentile. SMS: multiple sclerosis patients in a stable phase of disease; BT-AMS: AMS patients before the initiation of glucorticoids; DT-AMS: AMS patients after the initiation of glucorticoids; HC: healthy controls. SE: standard error;

* p<0.05 compared to HC;

^#^ p<0.05 compared to DT-AMS;

^¶^ p<0.05 compared to BT-AMS

Immune activation in the different conditions was evaluated by measuring neopterin levels as well. Results showed that serum neopterin concentration was increased, although not significantly, in BT-AMS (5.02±1.64 nM) compared to SMS (4.81±1.12 nM) and DT-AMS (4.70±1.10 nM) (Table 2).

The results obtained in DT-AMS individuals derive from analyses performed one day after initiation of GC; these results were similar to those seen when samples collected 1 or 4 days after initiation of therapy were analyzed (IDO fold: 0.06, 0.05–0.12, 1 day of treatment; 0.14, 0.09–0.58, 4 days of treatment; IFN-γ fold: 0.008, 0.003–0.013, 1 day of treatment, 0.006, 0.004–0.012, 4 days of treatment).

### IDO activity

**Fig 2:**
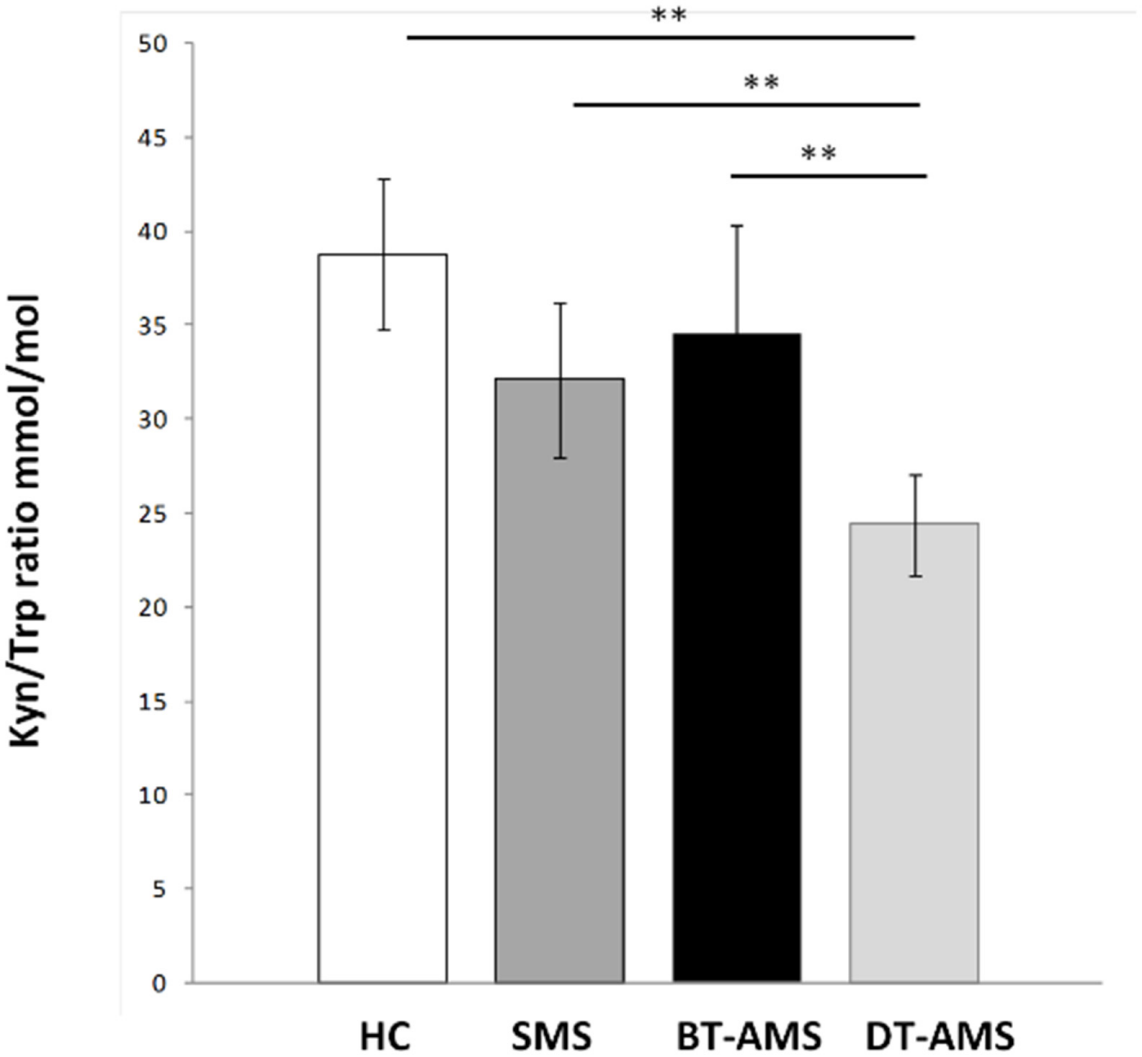
Kynurenine-to-Tryptophan ratio. Kyn/Trp in sera of multiple sclerosis patients with stable (SMS) or relapsing (AMS) disease and healthy controls (HC). AMS patients were analyzed before (BT-AMS) or after (DT-AMS) the initiation of glucocorticoid treatment. *p<0.05.

To evaluate IDO activity, the Kyn/Trp ratio was measured by HPLC in each individual. As shown in Fig 2 and summarized in Table 2, the Kyn/Trp ratio was decreased in SMS compared to HC, but this difference did not reach statistical significance. Interestingly, the Kyn/Trp ratio in BT-AMS was slightly augmented compared to SMS; this ratio was nevertheless significantly decreased by treatment (BT-AMS *vs*. DT-AMS: p<0.0001). Finally, the Kyn/Trp ratio was reduced in DT-AMS compared to SMS and HC (p<0.0001), whereas no differences were found between BT-AMS and HC. Again, no differences were seen in this parameter when results obtained 1, 2 or 4 days of therapy were compared (Kyn/Trp mmol/mol: 26.95±4.6, 1 day of treatment, 23.85±5.54, 4 days of treatment).

## Discussion

The biochemical processes leading to the catabolism of the essential amino acid Trp, *i*.*e*. the kynurenine pathway, play an important role in immune regulation. IDO, the key enzyme in this pathway, is ubiquitously expressed at low level in mammal cells, and cellular mechanisms responsible for its activation and regulation are not fully elucidated [21], although several its inducers are well-known, like IFN-γ and LPS [9, 22].

The demonstration that IDO expression plays a role in the remission of acute MS in the EAE animal model of MS suggested the possibility that this enzyme could be involved in regulating the clinical course of disease [12]; we thus decided to evaluated the expression profile of IDO and IFN-γ and the enzymatic activity of IDO (Kyn/Trp ratio) in correlation with clinical features in RR-MS patients. Moreover we measured the neopterin level in serum, a protein that reflects release of IFN-γ due to the immune system activation [23].

These parameters were analyzed in PBMCs and sera from patients during the clinical relapse of MS, before and during GC treatment. Data herein show that IDO and IFN-γ mRNA expression as well as serum concentration of neopterin change in patients with acute or stable MS. Thus, during disease relapses, IDO in lymphocytes and serum neopterin were increased and IFN-γ was greatly diminished compared to the values observed in the stable phases of disease. Ethical reasons prevented the possibility to analyze the spontaneous dynamic pattern of expression of target genes during the entire acute phase in patients. Data herein nevertheless show that GC-based therapy resulted in a prompt suppression not only of IFN-γ, in accordance with what was observed previously [24], but also of the expression of IDO. However, because obvious ethical reasons prevent us to withhold immunosuppressive therapy in patients in whom disease relapses occur, at least in theory it cannot be excluded that the observed modifications in IFN-γ and IDO levels occur naturally and spontaneously, and are not caused by GC.

In our analyses high levels of IDO expression as well as increased serum concentrations of neopterin were observed in RR-MS in concomitance with the appearance of clinical signs of disease relapse, indicating that an inflammatory response is indeed taking place in these patients. IFN-γ expression at the same time point was, on the other hand, down- regulated, in apparent contrast with previous results [24–27].

A possible explanation for the discrepancy between our data and those shown in [24–27] is that the peak of IFN-γ production precedes the clinical appearance of disease relapse. The biological process underlying disease activity does indeed precede clinical symptoms, as MRI results show that active lesions are detected before the clinical symptoms of MS relapse become evident [28]. Nevertheless, because MS is a Th1 and Th17 mediated disease, we cannot exclude that Th17 cytokines could suppress IFN-γ expression.

Our data nevertheless support recent results showing that discordant patterns of IDO and IFN-γ mRNA expression are present in lymph nodes, spinal cord and PBMCs through the preclinical, acute and remission phases of EAE [12].

The evaluation of IDO enzyme activity, calculated as Kyn/Trp ratio in serum, was a subsequent and necessary step for a correct interpretation of our results, as gene transcription is not sufficient to drive the enzyme activity. Surprisingly, analyses of the catalytic activity showed that the serum Kyn/Trp ratio was increased in MS patients undergoing disease relapse compared to what observed in the stable phases of MS, this activity was decreased upon initiation of GC, as expected given the known immunosuppressive effect of GC. Notably, GC resulted in a much more evident decrease of IDO gene expression compared to the Kyn/Trp ratio; this effect could be due to GC-mediated activation of other enzymes involved in Trp catabolism, such as tryptophan 2,3-dioxygenase (TDO) [29]. The observed modifications in gene expression and in the functional activity of IDO could, nevertheless, be also justified by post-translational modifications, the presence of natural IDO inhibitors (such as NO), the absence of co-factors (such as hemin), or, finally, structural and conformational aspects of substrate recognition [21, 30, 31].

IDO activation is suggested to be a self-protective response to alleviate autoaggressive CNS inflammation. This process results in immune suppression as a consequence of the depletion of Trp [32], and following the direct effect of catabolites (i.e. kynurenines) that inhibit the proliferation of auto-reactive T lymphocytes and increase their susceptibility to apoptosis [33]. Notably, IDO expression in dendritic cells promotes the conversion of naive T lymphocytes into Treg cells; such cells modulate immunotolerance and immunoregulation [34]. These observations led to the concept that an impairment in the IDO pathway could be involved in the development of autoimmune disease including MS, and resulted in the idea that modulation of IDO expression and activity could be beneficial in autoimmunity. Importantly, activation of Tregs was suggested to correlate with disease remission in MS thus reinforcing the speculation that IDO activity does play a role in modulating the clinical phenotype of this disease [35, 36]. A role for IDO depletion was suggested in the remission of symptoms in EAE [12] as reduced IFN-γ and increased IDO mRNA were observed in quiescent EAE, indicating that a higher enzyme activity could result in the depletion of auto-reactive and IFN-γ -producing T lymphocytes. A contribution of IDO to disease remission was also indicated by the relapses observed in mice after treatment with 1-methyl-thriptophane, an IDO inhibitor. These results raised the possibility that manipulating kynurenine pathway could be a way to treat MS relapse [12].

To our knowledge, this is the first in-depth evaluation of IDO expression and activity in PBMCs in relation to the clinical course of MS. It is important to underline that our results reflect the *in vivo* situation as they were obtained by evaluating mRNA expression in unstimulated lymphocytes. Ethical reasons did not allow following the behaviour of the IDO pathway during the relapsing phase, as GC therapy was initiated as soon as the patients were referred to the hospital. Notably, our results do not totally agree with those obtained in the animal model [12], probably because EAE model is not completely reproducible in humans.

In conclusion our data indicate that, in accordance with what was observed in the animal models, IDO could contribute to remission of relapse in MS. These findings raise the possibility that evaluation of IDO gene expression could be a useful predictive biomarker indicating the development of flares of disease, thus helping the promptly treatment of such flares, to reduce not only disease progression, but also related-disease cost and psychological effects for MS patients.
